# Supplementary material for: Topological phase locking in stochastic oscillators
Source: Nat Commun. 2025 May 24;16:4835. doi: 10.1038/s41467-025-60070-3 (PMC12103618; doi:10.1038/s41467-025-60070-3)
Supplement: Supplementary file 1 — Supplementary information [file 41467_2025_60070_MOESM1_ESM.pdf]

Topological phase locking in stochastic oscillators
Supplementary Information

Michalis Chatzittofi,[1] Ramin Golestanian,[1,2,*] and Jaime Agudo-Canalejo[1,3,†]

[1] *Max Planck Institute for Dynamics and Self-Organization (MPI-DS), D-37077 Göttingen, Germany*
[2] *Rudolf Peierls Centre for Theoretical Physics, University of Oxford, Oxford OX1 3PU, United Kingdom*
[3] *Department of Physics and Astronomy, University College London, London WC1E 6BT, United Kingdom*

(Dated: May 1, 2025)

CONTENTS

Supplementary Methods 2
Derivation of the phase equations 2

Supplementary Figures 3

Supplementary References 7

References 7

* ramin.golestanian@ds.mpg.de
† j.agudo-canalejo@ucl.ac.uk

SUPPLEMENTARY METHODS

Derivation of the phase equations

We consider two non-identical rotors that are close to each other or even attached. To describe their rotating dynamics, we use the angle θ_1 and θ_2 which correspond to the angle of rotor 1 and 2. These rotors can self-rotate thanks to a driven internal cyclic process, representing a chemical reaction. The dynamics of this internal process is described using the phases ϕ_1 and ϕ_2 which are the phases that appear in the main text.

Firstly, to describe the dynamics of a single rotor ($i = 1, 2$) we consider the potential

$$U_i(\theta_i, \phi_i) = -k_i \cos(n_i\theta_i - \phi_i) + V_i(\phi_i) \tag{1}$$

with $V_i(\phi_i)$ being the washboard potential described in the main text, $V_i(\phi_i) = -F_i\phi_i - v_i\cos(\phi_i + \delta_i)$. The first term represents a "toothed gear" potential which provides the mechanochemical coupling, with strength k_i, between the rotation angle θ_i and the chemical process described by ϕ_i. We remind that a chemical reaction corresponds to ϕ advancing by 2π. The integer n_i thus describes how many reactions it takes to complete a full turn of the rotor, with its sign defining the direction or chirality of the rotation (clockwise or anticlockwise). For example, for a typical ATP synthase which rotates 120° per reaction [1], we have $n_i = \pm 3$.

The surrounding medium or substrate causes hydrodynamic interactions between the two rotors and this induces a coupling between the two. Therefore the dynamical equations in terms of the torques τ_1 and τ_2 are,

$$\dot{\theta}_1 = \mu_{\theta 1}\tau_1 + g\tau_2 \tag{2}$$

$$\dot{\theta}_2 = g\tau_1 + \mu_{\theta 2}\tau_2 \tag{3}$$

where $\mu_{\theta i}$ are rotational hydrodynamic self-mobilities, and g is the coupling from hydrodynamic interactions (cross-mobility) or from friction due to direct contact. For example, for a rotating disk, $\mu_\theta = 1/(4\pi\eta a^2)$, with a the radius of the disk and η the hydrodynamic viscosity. Typically, the value g is negative, and in the case of two interacting rotating disks is $g = -1/(8\pi\eta r)$ with r the distance between the two rotors [2]. The torques τ_i are derived directly from the potential $U_i(\theta_i, \phi_i)$, as $\tau_i = -\partial_{\theta_i} U_i(\theta_i, \phi_i) = -k_i n_i \sin(n_i\theta_i - \phi_i)$.

The equations for the internal phases are also derived directly from the potential, as

$$\dot{\phi}_i = -\mu_{\phi i}\partial_{\phi_i} U_i(\theta_i, \phi_i) = \mu_{\phi i} k_i \sin(n_i\theta_i - \phi_i) - \mu_{\phi i} V_i'(\phi_i) \tag{4}$$

where $\mu_{\phi i}$ is the mobility governing the overdamped dynamics of the reaction coordinate ϕ_i. Let us denote $\delta\theta_i \equiv n_i\theta_i - \phi_i$. By assuming strong mechanochemical coupling, i.e. that the timescale of angle relaxation $(k_i\mu_{\theta i})^{-1}$ is much shorter than the timescale for the changes in internal phase velocity, we simplify the dynamics since the angles will relax quickly and the dynamics will be dictated by the dynamics of the phases ϕ_i, which are the slow variables. Mathematically, this is equivalent to $\delta\dot{\theta}_i \simeq 0$, which implies that $\dot{\theta}_i \simeq \dot{\phi}_i/n_i$. Substituting this into (2)-(3), we can solve for $k_1 \sin\delta\theta_1$ and $k_2 \sin\delta\theta_2$ and introduce them into (4). Further solving for $\dot{\phi}_1$ and $\dot{\phi}_2$, we finally obtain the deterministic part of Eq. (1) in the main text, i.e.

$$\dot{\phi}_1 = \mu_1[-V'(\phi_1)] + \sqrt{\mu_1\mu_2}\,h[-V'(\phi_2)] \tag{5a}$$

$$\dot{\phi}_2 = \sqrt{\mu_1\mu_2}\,h[-V'(\phi_1)] + \mu_2[-V'(\phi_2)] \tag{5b}$$

with the coefficients

$$\mu_1 \equiv \mu_{\phi 1}\left(1 + \frac{\mu_{\theta 1}\mu_{\phi 2}}{n_1 n_2(\mu_{\theta 1}\mu_{\theta 2} - g^2)}\right)\left(1 + \frac{\mu_{\theta 1}\mu_{\phi 2} + \mu_{\phi 1}\mu_{\theta 2} + \frac{\mu_{\phi 1}\mu_{\phi 2}}{n_1 n_2}}{n_1 n_2(\mu_{\theta 1}\mu_{\theta 2} - g^2)}\right)^{-1}, \tag{6}$$

$$\mu_2 \equiv \mu_{\phi 2}\left(1 + \frac{\mu_{\phi 1}\mu_{\theta 2}}{n_1 n_2(\mu_{\theta 1}\mu_{\theta 2} - g^2)}\right)\left(1 + \frac{\mu_{\theta 1}\mu_{\phi 2} + \mu_{\phi 1}\mu_{\theta 2} + \frac{\mu_{\phi 1}\mu_{\phi 2}}{n_1 n_2}}{n_1 n_2(\mu_{\theta 1}\mu_{\theta 2} - g^2)}\right)^{-1}, \tag{7}$$

$$h \equiv \frac{g}{n_1 n_2}\frac{\sqrt{\mu_{\phi 1}\mu_{\phi 2}}}{\mu_{\theta 1}\mu_{\theta 2} - g^2}\left(1 + \frac{\mu_{\theta 1}\mu_{\phi 2}}{n_1 n_2(\mu_{\theta 1}\mu_{\theta 2} - g^2)}\right)^{-\frac{1}{2}}\left(1 + \frac{\mu_{\phi 1}\mu_{\theta 2}}{n_1 n_2(\mu_{\theta 1}\mu_{\theta 2} - g^2)}\right)^{-\frac{1}{2}}. \tag{8}$$

The corresponding noise term in Eq. (1) of the main text follows directly from requiring a fluctuation-dissipation relation.

Additionally making the reasonable assumptions that the mobility of the internal phase is smaller than the hydrodynamic mobility of the angle ($\mu_{\phi i}/\mu_{\theta_i} \ll 1$), and that the hydrodynamic coupling is small ($g/\sqrt{\mu_{\theta_1}\mu_{\theta_2}} \ll 1$) we find that the coefficients to leading order in these ratios are

$$\mu_1 \simeq \mu_{\phi 1}, \quad \mu_2 \simeq \mu_{\phi 2}, \quad h \simeq \frac{g}{n_1 n_2} \frac{\sqrt{\mu_{\phi 1}\mu_{\phi 2}}}{\mu_{\theta 1}\mu_{\theta 2}}. \tag{9}$$

The sign of h depends on the signs of g, n_1, and n_2. Since in the case of hydrodynamic interactions g is negative, in order to obtain $h > 0$ we find that n_1 and n_2 must have opposite signs, that is, the rotors must rotate in opposite directions (with opposite chirality).

SUPPLEMENTARY FIGURES

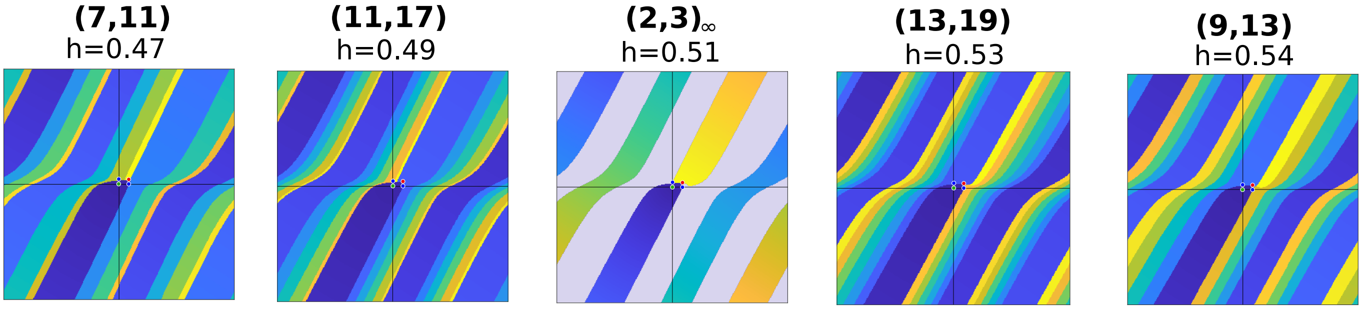

Supplementary Fig. 1. **Example of a transition.** Phase portraits as the system crosses through the $(2,3)_\infty$ TPL state with increasing h. The driving force asymmetry is $E_{*2}/E_{*1} = 19.15$. In all cases, $E_{\mathrm{ba}1}/E_{*1} = 3 \cdot 10^{-4}$.

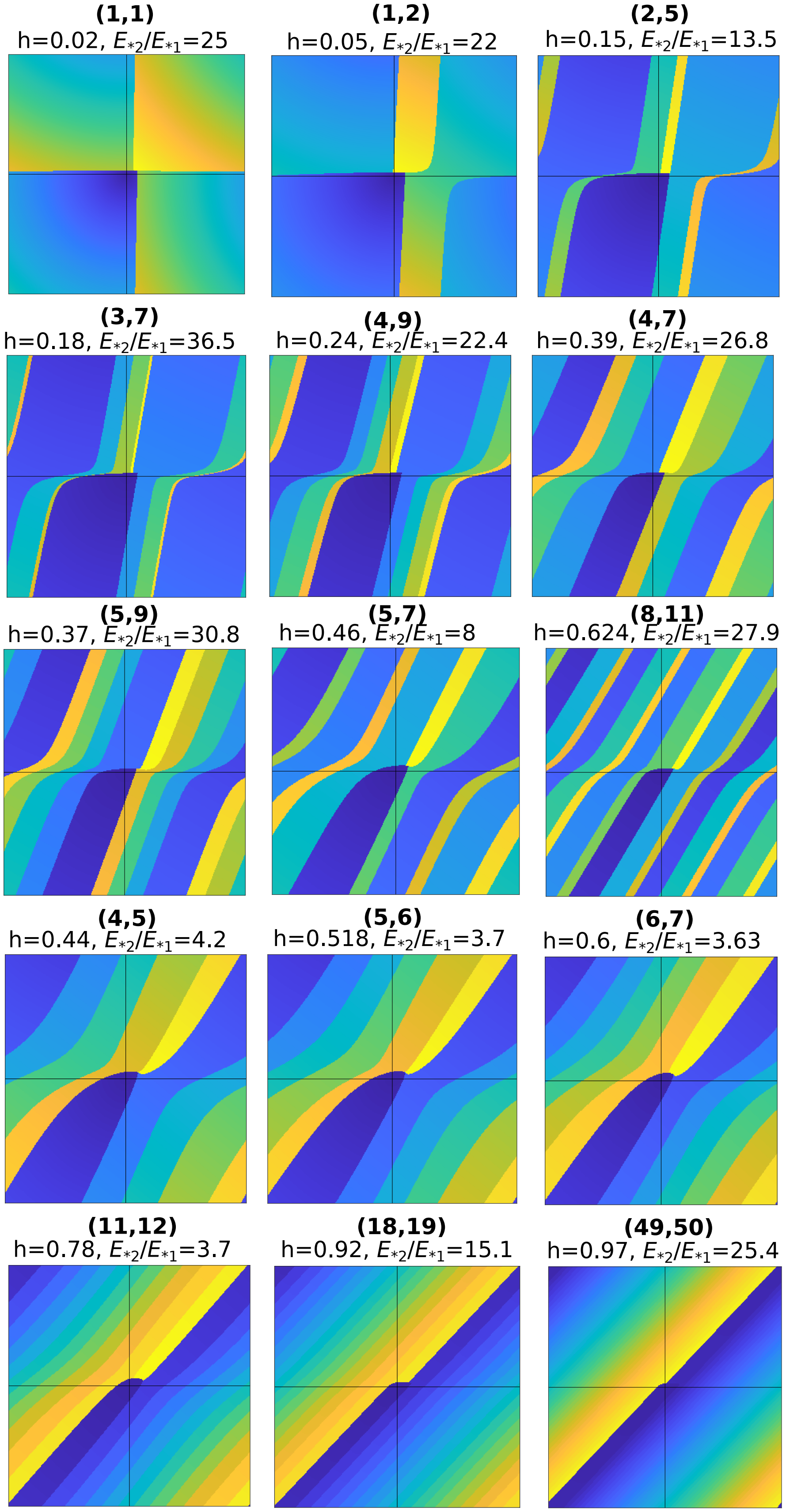

Supplementary Fig. 2. **Various phase portraits with finite phase locking.** In all cases, $E_{\mathrm{ba1}}/E_{*1} = 3 \cdot 10^{-4}$.

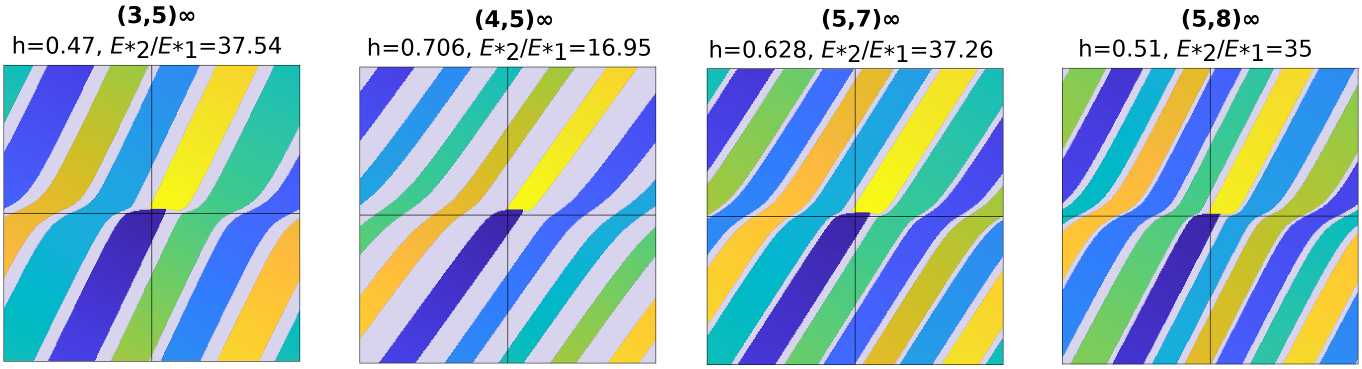

Supplementary Fig. 3. **Various TPL phase portraits.** For the topologies $(3,5)_\infty$, $(4,5)_\infty$ and $(5,7)_\infty$, we used $E_{ba1}/E_{*1} = 3 \cdot 10^{-4}$. For the portrait $(5,8)_\infty$, we used $E_{ba1}/E_{*1} = 1 \cdot 10^{-4}$.

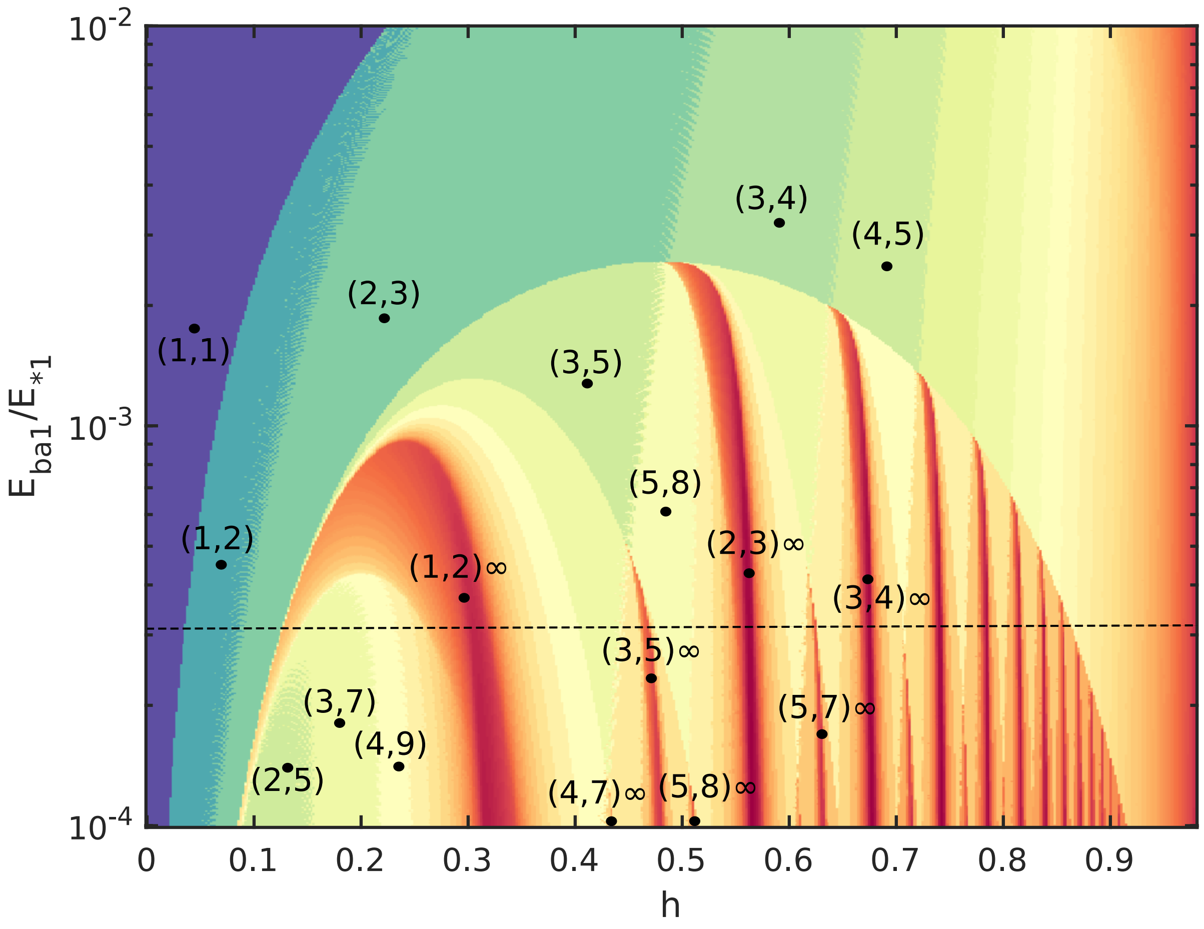

Supplementary Fig. 4. **Phase-locking diagram with fixed asymmetry.** The diagram as a function of coupling strength h and E_{ba1}/E_{*1}, for fixed $E_{ba2}/E_{ba1} = 1$ and $E_{*2}/E_{*1} = 35$. The horizontal line corresponds to $E_{ba1}/E_{*1} = 3 \cdot 10^{-4}$ which was used throughout the main text.

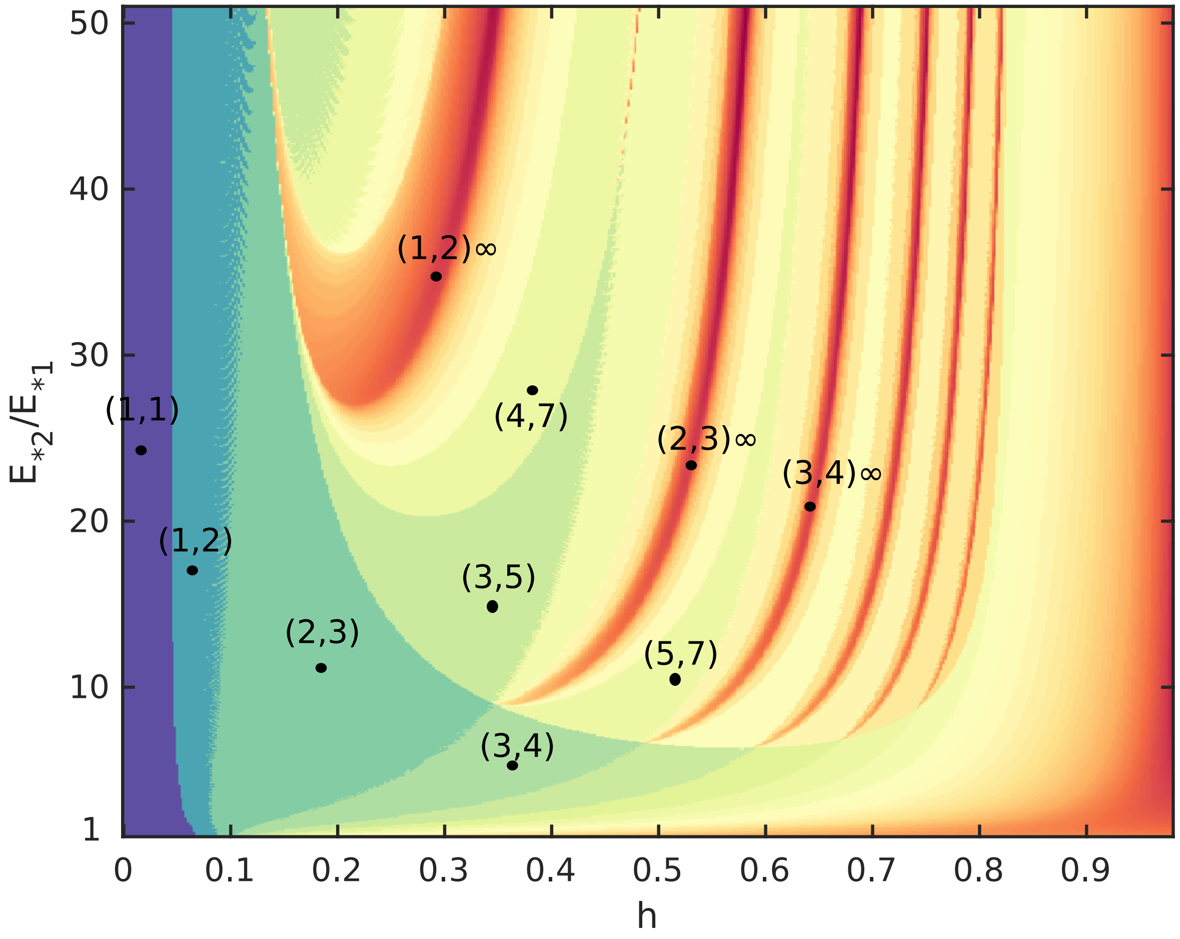

Supplementary Fig. 5. **Phase-locking diagram with double energetic asymmetry**. The diagram as a function of the coupling strength h and the driving force asymmetry E_{*2}/E_{*1}. In comparison with Fig. 5 in the main text, which used $E_{\mathrm{ba1}}/E_{*1} = 3 \cdot 10^{-4}$ and $E_{\mathrm{ba2}}/E_{\mathrm{ba1}} = 1$, here we use $E_{\mathrm{ba1}}/E_{*1} = 10^{-3}$ and $E_{\mathrm{ba2}}/E_{\mathrm{ba1}} = 0.5$.

SUPPLEMENTARY REFERENCES

[1] W. Junge and N. Nelson, ATP Synthase, Annu. Rev. Biochem. **84**, 631–657 (2015).
[2] S. K. Richter and A. M. Menzel, Mediated interactions between rigid inclusions in two-dimensional elastic or fluid films, Phys. Rev. E **105**, 014609 (2022).
