# Supplementary material for: Topological phase locking in stochastic oscillators
Source: Nat Commun. 2025 May 24;16:4835. doi: 10.1038/s41467-025-60070-3 (PMC12103618; doi:10.1038/s41467-025-60070-3)
Supplement: Supplementary file 2 — Description of additional supplementary files [file 41467_2025_60070_MOESM2_ESM.pdf]

Description of Additional Supplementary Files

File Name: Supplementary Movie 1

Description: An example of a stochastic (2,3) transition in a (3,4) finite phase locking topology. On the left panel, the evolution of the trajectory is shown on top of the phase portrait. On the right panel, the completed cycles are shown as a function of simulation time.

File Name: Supplementary Movie 2

Description: An example of the stochastic dynamics on a $(2,3)_\infty$ TPL topology. On the left panel, the evolution of the trajectory is shown on top of the phase portrait. On the right panel, the completed cycles are shown as a function of simulation time.
